# Putative EEG measures of social anxiety: Comparing frontal alpha asymmetry and delta–beta cross-frequency correlation

**DOI:** 10.3758/s13415-016-0455-y

**Published:** 2016-08-24

**Authors:** A. Harrewijn, M. J. W. Van der Molen, P. M. Westenberg

**Affiliations:** Developmental and educational psychology and Leiden Institute for Brain and Cognition, Leiden University, Leiden, The Netherlands

**Keywords:** Social anxiety, Biomarker, Alpha asymmetry, Delta–beta correlation, Social-performance task

## Abstract

The goal of the present study was to examine whether frontal alpha asymmetry and delta–beta cross-frequency correlation during resting state, anticipation, and recovery are electroencephalographic (EEG) measures of social anxiety. For the first time, we jointly examined frontal alpha asymmetry and delta–beta correlation during resting state and during a social performance task in high (HSA) versus low (LSA) socially anxious females. Participants performed a social performance task in which they first watched and evaluated a video of a peer, and then prepared their own speech. They believed that their speech would be videotaped and evaluated by a peer. We found that HSA participants showed significant negative delta–beta correlation as compared to LSA participants during both anticipation of and recovery from the stressful social situation. This negative delta–beta correlation might reflect increased activity in subcortical brain regions and decreased activity in cortical brain regions. As we hypothesized, no group differences in delta–beta correlation were found during the resting state. This could indicate that a certain level of stress is needed to find EEG measures of social anxiety. As for frontal alpha asymmetry, we did not find any group differences. The present frontal alpha asymmetry results are discussed in relation to the evident inconsistencies in the frontal alpha asymmetry literature. Together, our results suggest that delta–beta correlation is a putative EEG measure of social anxiety.

**Electronic supplementary material:**

The online version of this article (doi:10.3758/s13415-016-0455-y) contains supplementary material, which is available to authorized users.

Social anxiety disorder (SAD) is a common internalizing disorder that is characterized by extreme fear and avoidance of social situations (American Psychiatric Association, 2013). Cognitive–behavioral studies have shown that patients with SAD show information-processing biases during anticipation of and recovery from stressful social situations. For example, patients with SAD more often recall negative information about themselves during anticipation of a stressful situation, expect more negative outcomes of social situations, and show protracted postevent processing (Clark & McManus, 2002; Hirsch & Clark, 2004). A variety of studies have used social performance tasks to examine the electrophysiological correlates of these information-processing biases in response to stressful social situations (Davidson, Marshall, Tomarken, & Henriques, 2000; Miskovic et al., 2010; Miskovic, Campbell, et al., 2011; Miskovic, Moscovitch, et al., 2011). These studies typically compare electrophysiological reactivity during baseline (resting state) with an anticipation phase in which participants are about to experience a stressful social situation. Using social performance tasks, electrophysiological investigations have shown promising results, due to their high temporal precision in capturing objective brain reactivity measures during various stages of processing stressful social situations (Amodio, Bartholow, & Ito, 2014; Kotchoubey, 2006; Luck, 2005). This line of work has led to two putative electroencephalographic (EEG) measures of social anxiety that may aid in the early detection, prevention, and treatment of SAD: frontal alpha asymmetry and delta–beta cross-frequency correlation (further referred to as the *delta–beta correlation*). The goal of the present study was to validate these putative EEG measures of social anxiety by a direct comparison of frontal alpha asymmetry and delta–beta correlation in different phases of a social performance task.

An influential theoretical account proposed by Davidson (1992, 1998) claims that individuals who display relatively stronger left frontal cortical activity are biased toward approach-related behavior, whereas individuals who display relatively stronger right frontal cortical activity are biased toward withdrawal-related behavior (Davidson, 1992, 1998). This hemispheric lateralization of brain activity related to approach versus avoidance behavior is reflected in frontal alpha power (8–13 Hz) asymmetry metrics (Allen, Coan, & Nazarian, 2004; Davidson, 1992, 1998). Frontal alpha asymmetry is typically measured by subtracting the log-transformed left-lateralized frontal alpha power from the log-transformed right-lateralized frontal alpha power (Allen et al., 2004). Since alpha power is inversely related to cortical activity, positive alpha asymmetry scores reflect relatively greater left frontal cortical activity (i.e., decreased left frontal alpha power), and negative alpha asymmetry scores reflect relatively greater right frontal cortical activity (i.e., decreased right frontal alpha power; Allen et al., 2004). Several reviews have shown that relatively greater right frontal cortical activity serves as a moderator for the development of various internalizing disorders and is related to behavioral inhibition (Coan & Allen, 2003, 2004), a temperamental style relevant to the etiology of SAD (Clauss & Blackford, 2012).

Frontal alpha asymmetry research in social anxiety has been limited, but some evidence has suggested that high socially anxious (HSA), high socially withdrawn, and shy participants display relatively increased right frontal cortical activity during resting-state EEG (Campbell et al., 2007; Hannesdottir, Doxie, Bell, Ollendick, & Wolfe, 2010; Schmidt, 1999). The consistency of this finding has been questioned, because others have failed to replicate this pattern of relatively increased right frontal cortical activity during resting state in social anxiety (Beaton et al., 2008; Cole, Zapp, Nelson, & Perez-Edgar, 2012; Davidson et al., 2000). Findings of frontal alpha asymmetry during anticipation of stressful social situations have also been mixed. Davidson et al. (2000) reported relatively elevated right frontal cortical activity in patients with SAD as compared to controls, and Cole et al. (2012) showed relatively increased right frontal cortical activity in high versus low socially withdrawn participants, but only during a highly stressful condition—namely, after viewing a video of a peer talking in an embarrassed and anxious way about a past embarrassing moment, right before participants themselves had to prepare their own speech. However, Beaton et al. (2008) did not find differences in frontal alpha asymmetry between HSA and low socially anxious (LSA) participants while anticipating a speech task. Most studies have focused on frontal alpha asymmetry during anticipation of a stressful social event, but cognitive–behavioral studies have shown that HSA participants also show information-processing biases during recovery from a stressful social event (such as postevent rumination; Brozovich & Heimberg, 2008). Only one study has focused on frontal alpha asymmetry patterns during recovery from a social stressor, but no differences were found between patients with SAD and controls (Davidson et al., 2000). Together, these studies have provided mixed evidence that frontal alpha asymmetry can be considered an electrophysiological measure of social anxiety, either during resting state or when confronted with a social stressor.

Besides frontal alpha asymmetry, the association between delta (1–4 Hz) and beta (14–30 Hz) power (i.e., *delta–beta correlation*) has also been interpreted as a putative EEG measure of social anxiety. Several studies have shown that a positive delta–beta correlation is increased in anxiogenic situations (for a review, see Schutter & Knyazev, 2012). It has been suggested that slow-wave oscillations in the delta frequency range stem from subcortical regions, whereas fast-wave oscillations in the beta frequency range stem from cortical regions (Schutter & van Honk, 2005). Significant positive delta–beta correlation has been interpreted to reflect the crosstalk between cortical and subcortical regions (Miskovic, Campbell, et al., 2011; Miskovic, Moscovitch, et al., 2011; Putman, Arias-Garcia, Pantazi, & van Schie, 2012; Schutter & Knyazev, 2012; Schutter, Leitner, Kenemans, & van Honk, 2006; Schutter & van Honk, 2005; Velikova et al., 2010). Typically, significant positive delta–beta correlation is associated with anxiety. For example, significant positive delta–beta correlation is related to more attention to threat in an emotional Stroop task (Putman et al., 2012). In an anxiogenic situation, positive delta–beta correlation increases activation in a cortical network comprising the orbitofrontal cortex and anterior cingulate cortex. An increase in delta–beta correlation is associated with an increase of delta power and connectivity in these cortical regions (Knyazev, 2011). Together, these studies have revealed that anxiety in general is related to significant positive delta–beta correlation, whereas no correlation between delta and beta is related to a more relaxed state.

The possibility of delta–beta correlation as a putative EEG measure of social anxiety during resting-state EEG has been demonstrated by Miskovic, Campbell, et al. (2011) and Miskovic, Moscovitch, et al. (2011). These authors reported that patients with SAD showed significant positive delta–beta correlation before cognitive–behavioral therapy and no delta–beta correlation after therapy (Miskovic, Moscovitch, et al., 2011), and that children of a parent with SAD showed significant positive delta–beta correlation relative to typically developing children (Miskovic, Campbell, et al., 2011). Obviously, these results should be interpreted with caution, because these studies only reported on resting-state data, whereas others have shown that delta–beta correlation is increased only in an anxious state (Schutter & Knyazev, 2012). Moreover, the latter study was based on a small sample size (*n* = 6). However, delta–beta correlation does seem to be relevant in the pathophysiology of social anxiety, because significant positive delta–beta correlation has been found in HSA as compared to LSA individuals during anticipation of giving a speech in front of a camera (Miskovic et al., 2010). This significant positive delta–beta correlation was associated with higher levels of self-reported nervousness, less confidence, less calmness, less preparedness, and poorer estimates of the anticipated speech performance (Miskovic et al., 2010). Thus, although few studies have investigated delta–beta correlation during either resting state or anticipation, preliminary evidence suggests that significant positive delta–beta correlation during resting state and anticipation could be an EEG measure of social anxiety (Miskovic et al., 2010; Miskovic, Campbell, et al., 2011; Miskovic, Moscovitch, et al., 2011).

Due to the mixed results about these putative measures in prior investigations, the main objective of this study was to validate whether frontal alpha asymmetry and delta–beta correlation can be considered electrophysiological measures of social anxiety. Moreover, we included three phases of examination for each participant (i.e., resting state, anticipation, and recovery), which allowed for determining whether group differences in frontal alpha asymmetry and delta–beta correlation can be detected as trait (i.e., during the resting state) or as state (i.e., during anticipation of and/or recovery from a social stressor) phenomena. Notably, electrophysiological studies have focused mostly on anticipation and have shown inconsistent results. Thus, the present inclusion of a recovery phase could yield a better understanding of the state-versus-trait characteristics of these alleged EEG measures, as well as their temporal relevance in biased information processing in social anxiety (i.e., biased anticipatory attention vs. ruminative thinking during recovery from a social stressor). Similar to previous EEG studies, we used a social performance task to elicit the arousal associated with social performance anxiety (Clark & McManus, 2002; Heinrichs & Hofmann, 2001; Westenberg et al., 2009). In our version of the social performance task, HSA and LSA participants watched and evaluated a video of a peer before preparing their own speech that would be videotaped and evaluated by a peer. The unique aspect of this study is that we focused on both frontal alpha asymmetry and delta–beta correlation—measured in the same participants—during all three phases of the experiment. We hypothesized that if right frontal cortical activity and positive delta–beta correlation are EEG measures of social anxiety, these measures would be increased in HSA relative to LSA participants. Although the results from previous studies have been inconsistent, we used the theoretical background to hypothesize in which phase these putative EEG measures would be present. First, since increased right frontal cortical activity (as measured via frontal alpha asymmetry) is related to avoidance-related behavior (Davidson, 1992, 1998), we hypothesized that this would be present only during anticipation, not during resting state or recovery, because of the mixed findings in resting-state studies and because avoidance is not possible after the stressful social event. Second, because significant positive delta–beta correlation is related to crosstalk between cortical and subcortical regions in an anxious state (Schutter & van Honk, 2005), we hypothesized that this could be present during both anticipation and recovery, and not during the resting state.

## Method

### Participants

Participants were selected from 386 female undergraduate students who completed self-reports of the Liebowitz Social Anxiety Scale (LSAS; Liebowitz, 1987). Because studies about gender differences in frontal alpha asymmetry have shown inconsistent results (Jesulola et al., 2015), we included only female participants in order to reduce interindividual variability. On the basis of their LSAS scores, participants were assigned to either an LSA group (LSAS score < 30) or an HSA group (LSAS score ≥ 60). These cutoff scores were based on results from Mennin et al. (2002) indicating that LSAS scores lower than 30 are not associated with clinical social anxiety, whereas LSAS scores of 60 and higher are associated with generalized SAD. We administered the LSAS first at screening and again after the experiment to validate that the participants were still high or low socially anxious during the experiment. We excluded participants who showed an extreme difference (greater than 2 *SD*s) in LSAS score between screening and testing. The correlation between the LSAS scores at screening and during testing was high (*τ* = .66, *p* < .001). Three HSA participants were excluded due to data acquisition problems (*n* = 1), extreme differences between LSAS scores during screening and testing (*n* = 1), and unwillingness to participate in the social-performance task (*n* = 1). Two LSA participants were excluded due to an extreme difference between LSAS scores (*n* = 1) and missing questionnaire data (*n* = 1). This resulted in a final sample of 23 HSA (mean age = 19.56 years, *SD* = 1.43) and 33 LSA (mean age = 19.81, *SD* = 1.45) female participants. Age did not differ between the groups, *F*(1, 54) = .41, *p* = .53, *η* ^*2*^ = .01.

All participants were healthy, free from psychoactive medication, and right-handed, as confirmed with the Edinburgh Handedness Inventory (Oldfield, 1971), and had normal or corrected-to-normal vision. Participants were recruited from or within the proximity of Leiden University and were rewarded with €17 or course credit for their participation. All participants provided signed informed consent. This procedure is according to the Declaration of Helsinki. The ethics committee of the Institute of Psychology of Leiden University reviewed and approved this study.

### Procedure

Participants first received an explanation about the EEG procedure and signed the informed-consent form. After we attached the electrodes, the EEG protocol started with measuring EEG resting state for 5 min (eyes closed). Thereafter, participants performed a social judgment task (these data are reported elsewhere) and the social performance task. Finally, participants filled out the questionnaires and were debriefed. The experiment took 2.5 h in total.

### Social performance task

**Fig. 1:**
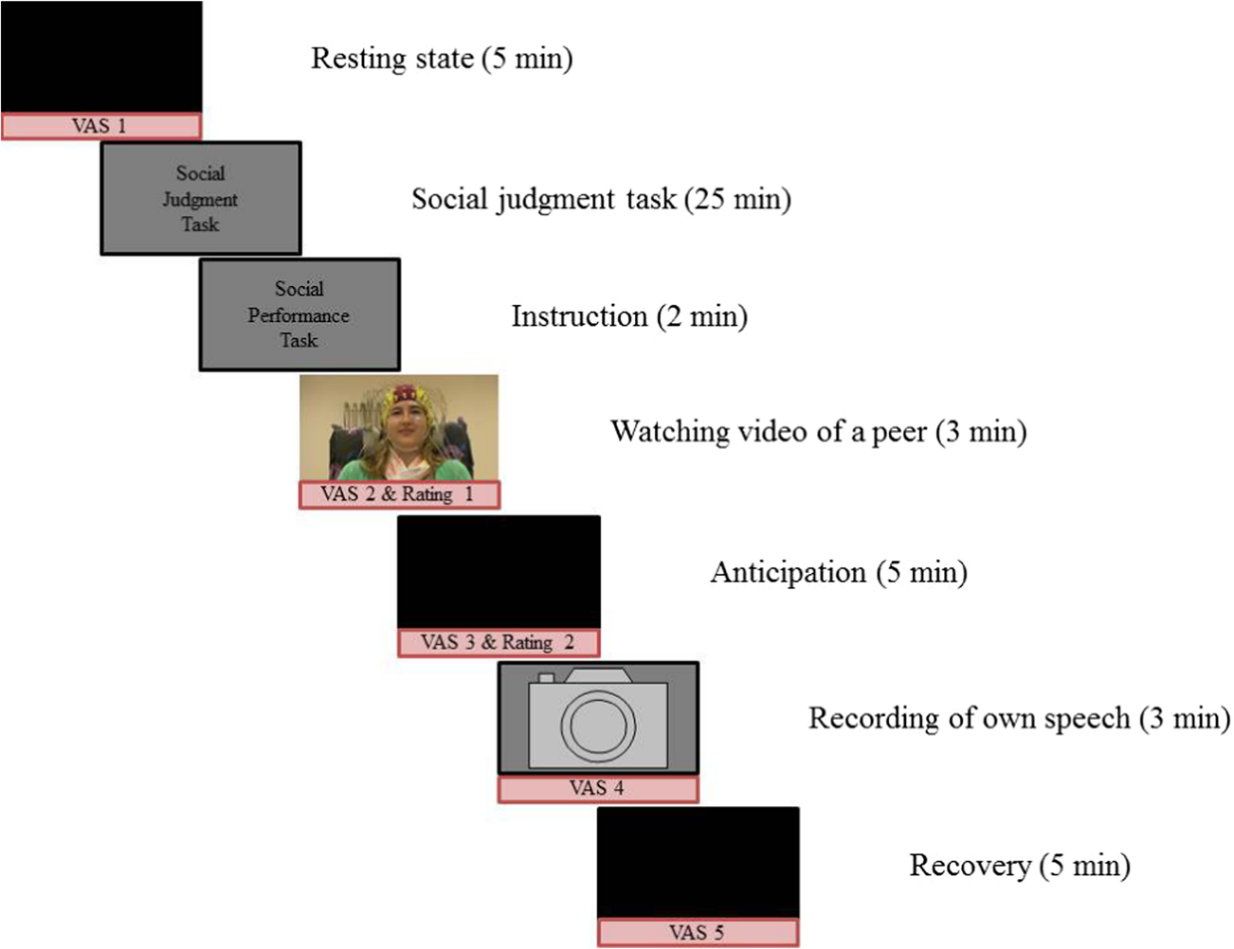
Overview of the experiment. EEG was recorded during resting state, anticipation, and recovery. The results of the social judgment task will be reported elsewhere

To measure EEG activity during a stressful social situation, we used a modified version of the social performance task presented in Rinck et al. (2013). Our social performance task comprised five phases (instruction, video, anticipation, speech, and recovery), which were presented in a fixed order and are depicted in Fig. 1. First, participants were informed about the task, because they did not know beforehand about this task. This was done to avoid anticipatory stress during resting-state EEG that was collected prior to the social performance paradigm. We explained to participants that they would view and judge a video of a peer telling about her positive and negative characteristics (Rating 1; see Supplementary Data 1 for these results). Then, participants prepared a speech about their own positive and negative characteristics (anticipation). Using a cover story, we explained that their video would be shown to a peer, and that this peer would judge the participant’s video (this was not the case). Participants were asked how they thought their video would be judged by a peer (Rating 2; see Supplementary Data 1 for these results). After participants had given their speech for 3 min in front of a camera, they had 5 min to relax (recovery). All participants reported after the experiment that they believed the cover story.

### Task-related nervousness and avoidance

At five time points during the social performance task (see Fig. 1), we asked participants to indicate how nervous they felt on a visual analogue scale (VAS) from 0 (*not at all*) to 100 (*very much*), and how much they felt like doing the next part of the experiment on a VAS from 0 (*not at all*) to 100 (*very much*). The latter question was used to indirectly measure avoidance, because in our view it was not ethical to ask participants five times whether they wanted to avoid a situation and do nothing about it.

### Self-report questionnaires

Social anxiety was measured with the LSAS (Liebowitz, 1987), which consists of 24 social situations. Participants rated on a 4-point Likert scale their anxiety (0 = *none*, 3 = *severe*) and avoidance (0 = *never*, 3 = *usually*) in each of these situations in the last week. The LSAS has a high internal consistency (*α* > .90; Liebowitz, 1987). In addition, to validate the HSA and LSA groups on the basis of social-anxiety-related constructs, we furthermore administered questionnaires that indexed fear of negative evaluation (Bögels & Reith, 1999), fear of positive evaluation (Weeks, Heimberg, & Rodebaugh, 2008), posttask rumination (Edwards, Rapee, & Franklin, 2003; Miers, Blote, Heyne, & Westenberg, 2014), and depression (Beck, Steer, Ball, & Ranieri, 1996).

### EEG recording and signal processing

EEG was measured with 64 Ag–AgCl electrodes mounted in an elastic electrode cap (10–20 placement) using the BioSemi Active Two system (Biosemi, Amsterdam, The Netherlands). EEG was recorded with a sampling rate of 1024 Hz. The common mode sense (CMS) and driven right leg replaced the conventional ground electrode, and CMS was used as an online reference. Vertical ocular movements were measured with electrodes placed above and below the left eye. Horizontal ocular movements were measured with electrodes placed at the left and right canthi. Two electrodes were placed at the left and right mastoid for offline referencing. We additionally measured the electrocardiogram via the modified lead-2 placement, but these data will be reported elsewhere.

EEG time series were analyzed with Brain Vision Analyzer (Brain Products GmbH). The EEG channels were re-referenced to the average of all EEG electrodes^1^ and filtered between 0.1 and 50 Hz (24 dB/oct), with a 50-Hz notch filter. Epochs of 4 s (4,096 samples) were created with 1 s (1,024 samples) overlap, and manually inspected for artifacts. Noisy channels were interpolated, and eye movements were subtracted from the data with ocular independent component analysis, as implemented in BVA. Epochs were automatically excluded on the basis of the following criteria: maximal allowed voltage step, 50 *μ*V/ms; minimum/maximum amplitude, –200/200 *μ*V; lowest allowed activity in 100-ms intervals, 0.5 *μ*V. If an artifact was found in one channel, the whole segment was removed during both manual and automatic artifact rejection. HSA and LSA participants did not differ in their numbers of clean epochs per phase of the task (resting state, anticipation, and recovery), all *p*s > .05 (see Table 1). Finally, we ran a fast Fourier transform analysis with a 50 % Hanning window to extract relative power (*μ*V^2^) from the delta (1–4 Hz),^2^ alpha (8–13 Hz), total-beta (14–30 Hz), low-beta (14–20 Hz), and high-beta (20–30 Hz) frequency bands. With respect to beta power, we distinguished between high and low beta power in order to examine the contributions of high and low beta separately to the delta–beta correlation results, and to allow for a better comparison of our study with those that have used only high or low beta bands (Miskovic et al., 2010; Miskovic, Moscovitch, et al., 2011).

**Table 1:** Mean, standard deviation (*SD*), and range of the numbers of clean epochs per phase in the social performance task (resting state, anticipation, and recovery), per group

		Mean	*SD*	Minimum	Maximum
Resting state	HSA	88.83	8.70	68	99
	LSA	91.64	5.04	79	99
Anticipation	HSA	87.13	12.05	49	98
	LSA	86.97	8.87	60	98
Recovery	HSA	90.30	10.55	58	99
	LSA	91.09	9.07	54	99

We focused on relative power values, because relative power better reflects cortical activity (Allen et al., 2004; Cook, O’Hara, Uijtdehaage, Mandelkern, & Leuchter, 1998) and is less confounded by scalp thickness and electrical resistance than is absolute power (Allen et al., 2004; Knyazev, Savostyanov, & Levin, 2004). The use of relative power decreases data variability and increases the probability of finding relationships between EEG and personality (Knyazev, 2007; Knyazev et al., 2004). The power estimates from all spectral bands were averaged across trials and log-transformed to obtain normal distributions.

### Frontal alpha asymmetry

Because previous studies led to inconsistent results with regard to the localization of peak alpha asymmetry scores, we computed composite alpha scores based on the average of alpha power in a left (F3, F5) and right (F4, F6) frontal cluster. Moreover, this approach avoids an arbitrary choice of electrode pairs. Alpha asymmetry values were obtained within participants by subtracting the left frontal alpha power from the right frontal alpha power (ln[right] – ln[left]), which corrects for overall alpha power levels and reduces individual differences related to skull thickness (Allen et al., 2004).

### Delta–beta correlation

The power values for electrodes F3, Fz, and F4 were averaged into composite frontal delta and beta power values (Putman, 2011; Putman et al., 2012). Kendall’s tau correlations were computed between delta and beta power separately for each group (HSA and LSA) in each condition (resting state, anticipation, recovery) for total beta, low beta, and high beta power separately. We used this between-subjects measure of delta–beta correlation to directly compare our findings with previous studies on social anxiety (Miskovic et al., 2010; Miskovic, Campbell, et al., 2011; Miskovic, Moscovitch, et al., 2011). In addition, to be able to consider individual differences, we computed a within-subjects measure of delta–beta correlation (see Supplementary Data 2).

### Statistical analysis

The analyses were performed in three steps to examine (1) self-report data, (2) frontal alpha asymmetry, and (3) delta–beta correlation. First, we analyzed self-report data to validate the groups and the social performance task. Group differences in the questionnaires were examined using analyses of variance (ANOVAs) and Mann–Whitney tests for variables that were not normally distributed. We examined group differences in nervousness and avoidance during the social performance task using nonparametric Mann–Whitney tests per time point, since these variables were not normally distributed. We applied a Bonferroni correction (*α* = .01) to correct for multiple comparisons. Second, we analyzed frontal alpha asymmetry during (a) resting state and (b) the social performance task, using nonparametric Mann–Whitney tests, since the log-transformed alpha asymmetry scores were not normally distributed. Third, we analyzed delta–beta correlation during (a) resting state and (b) the social performance task. We examined differences between groups in Kendall’s *τ* correlation coefficients using a Fisher’s *r*-to-*Z* transformation for resting state and the social performance task separately. For all analyses, we used IBM SPSS Statistics 21 (IBM Corp., Armonk, NY) and set alpha at .05. Greenhouse–Geisser corrections were used whenever appropriate, but uncorrected degrees of freedom were reported for transparency.

## Results

### Behavioral data

#### Self-report questionnaires

Table 2 shows the means and standard deviations of the HSA and LSA participants on the questionnaires. As compared to LSA participants, HSA participants displayed significant higher levels of social anxiety (during screening and after the experiment), depression, fear of negative evaluation, fear of positive evaluation, and negative rumination (all *p*s < .001).

**Table 2:** Overview of mean (SD) social anxiety, depression, fear of negative evaluation, fear of positive evaluation, and rumination scores in HSA and LSA participants

	HSA (*n* = 23)	LSA (*n* = 33)	*U*	*z*	*p* value	*r*
LSAS (screening)	73.13 (10.98)	19.18 (7.72)	759.00	6.33	<.001	.42
LSAS (testing)	75.35 (18.87)	25.33 (12.19)	756.50	6.28	<.001	.42
Depression (BDI)	12.22 (6.71)	6.45 (4.78)	590.00	3.52	<.001	.24
Rumination (positive)	7.04 (4.47)	8.67 (5.69)	333.00	–.78	.44	–.05
			*F*		*p* value	*η* ^*2*^
Fear of negative evaluation (FNE)	31.61 (8.66)	18.76 (11.40)	20.81		<.001	.28
Fear of positive evaluation (FPES)	37.83 (10.85)	21.18 (13.63)	23.77		<.001	.31
Rumination (negative)	27.57 (9.27)	13.27 (9.04)	33.16		<.001	.38

#### Nervousness

**Fig. 2:**
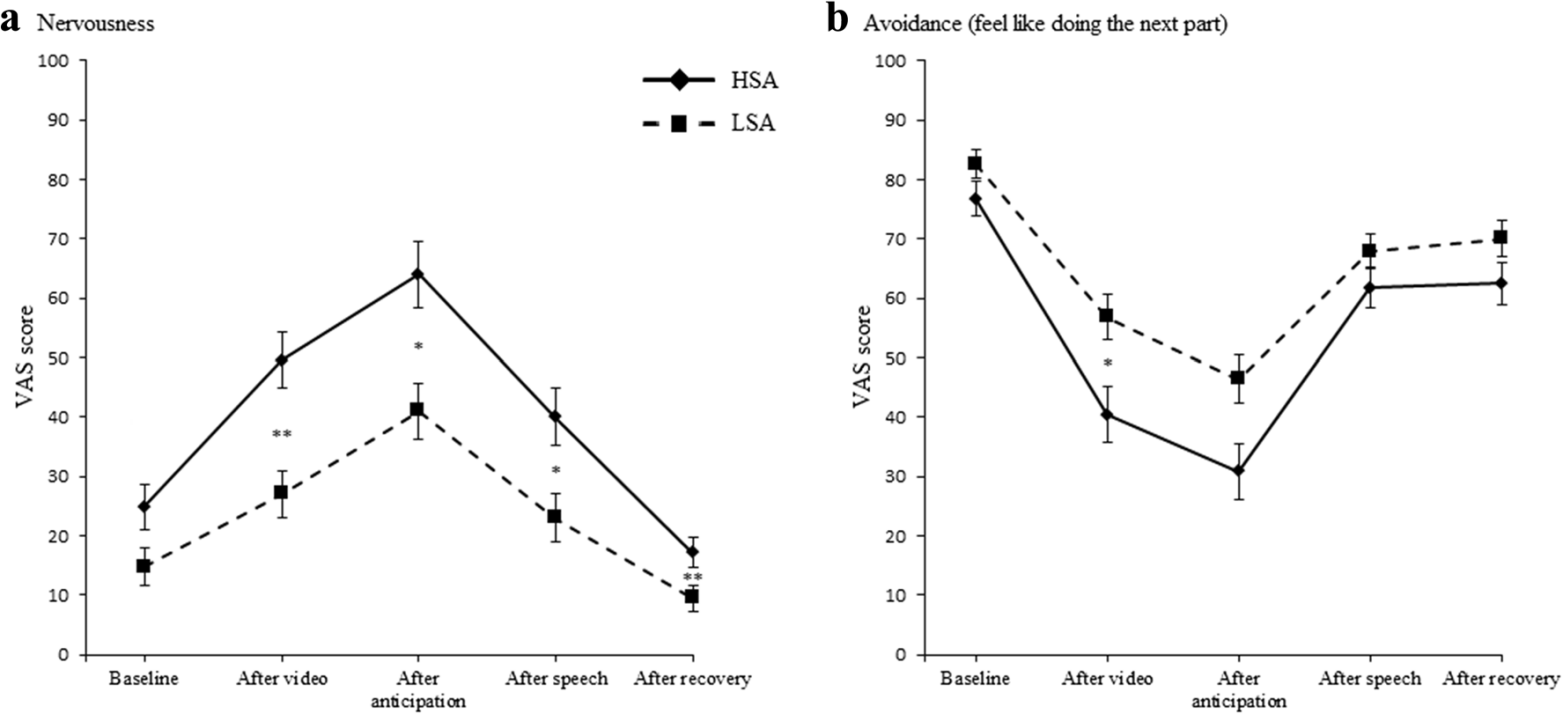
Nervousness (a) and avoidance (b) during the social performance task (^*^ *p* < .01, ^**^ *p* < .002; error bars represent standard errors). High socially anxious (HSA) participants showed more nervousness during the social performance task and avoidance after the video than did low socially anxious (LSA) participants

We compared nervousness between HSA and LSA participants at five time points during the social performance task (Fig. 2a). After Bonferroni correction (*α* = .01), there was no difference between HSA and LSA participants at baseline, *U* = 529.00, *z* = 2.50, *p* = .012, *r* = .15. HSA were more nervous than LSA participants at all of the other time points: respectively, *U* = 576.00, *z* = 3.27, *p* = .001, *r* = .20; *U* = 540.00, *z* = 2.67, *p* = .008, *r* = .16; *U* = 560.50, *z* = 3.02, *p* = .003, *r* = .18; *U* = 575.50, *z* = 3.27, *p* = .001, *r* = .20.

#### Avoidance

After Bonferroni correction (*α* = .01), we observed no difference between HSA and LSA participants at baseline, *U* = 281.50, *z* = –1.63, *p* = .102, *r* = –.10. After the video, HSA participants felt less like doing the task than did LSA participants, *U* = 225.50, *z* = –2.57, *p* = .010, *r* = –.15. During the rest of the task, there was no difference between HSA and LSA participants: respectively, *U* = 237.50, *z* = –2.37, *p* = .02, *r* = –.14; *U* = 319.00, *z* = –1.01, *p* = .31, *r* = –.06; *U* = 289.00, *z* = –1.51, *p* = .13, *r* = –.09.^3^

### Frontal alpha asymmetry

#### Resting state

**Fig. 3:**
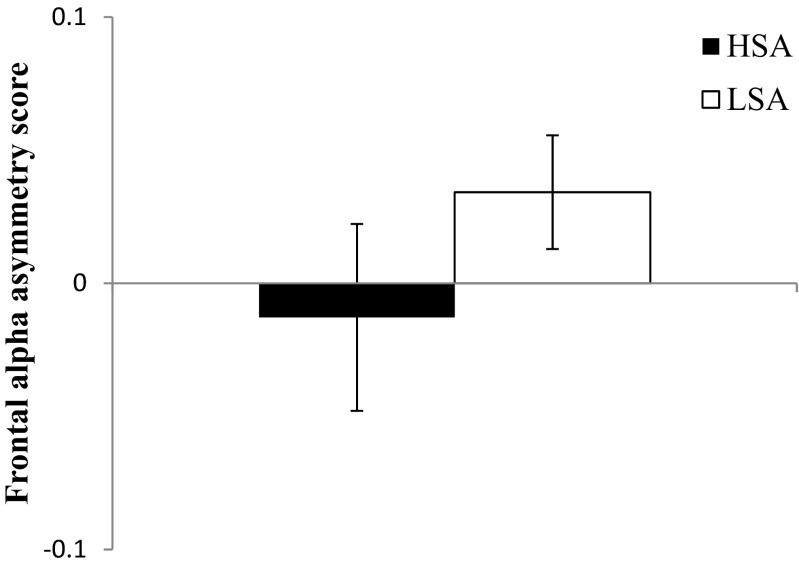
Frontal alpha asymmetry scores for HSA and LSA participants during resting state. Error bars represent standard errors

As we hypothesized, no difference was apparent between HSA and LSA participants in frontal alpha asymmetry during resting state, *U* = 289.00, *z* = –1.51, *p* = .13, *r* = –.12 (Fig. 3).

#### Social-performance task

**Fig. 4:**
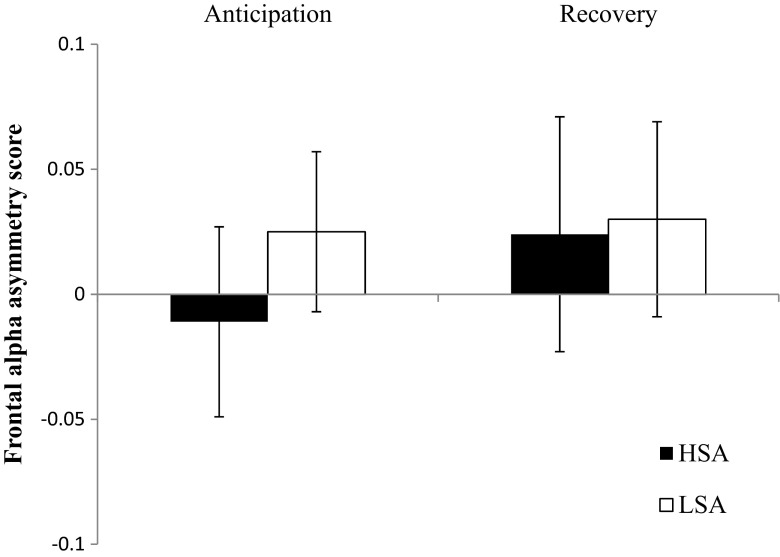
Frontal alpha symmetry scores for HSA and LSA participants during anticipation and recovery. Error bars represent standard errors

HSA and LSA participants did not differ in frontal alpha asymmetry during either anticipation or recovery: respectively, *U* = 334.00, *z* = –.76, *p* = .45, *r* = –.06; *U* = 359.00, *z* = –.34, *p* = .73, *r* = –.03 (Fig. 4).

### Delta–beta cross-frequency correlation

Table 3 shows absolute and relative delta and beta power across the groups and phases of the social-performance task.

**Table 3:** Absolute and relative delta and beta power (log-transformed) for the frontal cluster in HSA and LSA participants during resting state (RS), anticipation (ANT), and recovery (REC)

		HSA		LSA	
		Absolute	Relative	Absolute	Relative
RS	Delta (1–4 Hz)	–.67 (.08)	.55 (.05)	–.57 (.06)	.46 (.07)
	Beta (14–30 Hz)	–2.93 (.12)	–1.69 (.06)	–2.91 (.10)	–1.86 (.06)
	Low beta (14–20 Hz)	–2.57 (.13)	–1.34 (.08)	–2.56 (.11)	–1.52 (.06)
	High beta (20–30 Hz)	–3.24 (.11)	–2.00 (.05)	–3.22 (.09)	–2.16 (.07)
ANT	Delta (1–4 Hz)	–.63 (.09)	.70 (.04)	–.53 (.07)	.66 (.04)
	Beta (14–30 Hz)	–2.64 (.13)	–1.28 (.06)	–2.54 (.11)	–1.33 (.05)
	Low beta (14–20 Hz)	–2.45 (.13)	–1.09 (.06)	–2.37 (.11)	–1.16 (.04)
	High beta (20–30 Hz)	–2.81 (.13)	–1.46 (.08)	–2.68 (.12)	–1.47 (.07)
REC	Delta (1–4 Hz)	–.84 (.08)	.64 (.05)	–.86 (.07)	.61 (.04)
	Beta (14–30 Hz)	–2.76 (.13)	–1.09 (.08)	–2.92 (.11)	–1.19 (.06)
	Low beta (14–20 Hz)	–2.62 (.12)	–1.12 (.06)	–2.75 (.10)	–1.26 (.04)
	High beta (20–30 Hz)	–2.90 (.14)	–1.40 (.09)	–3.06 (.11)	–1.57 (.07)

#### Resting state

**Fig. 5:**
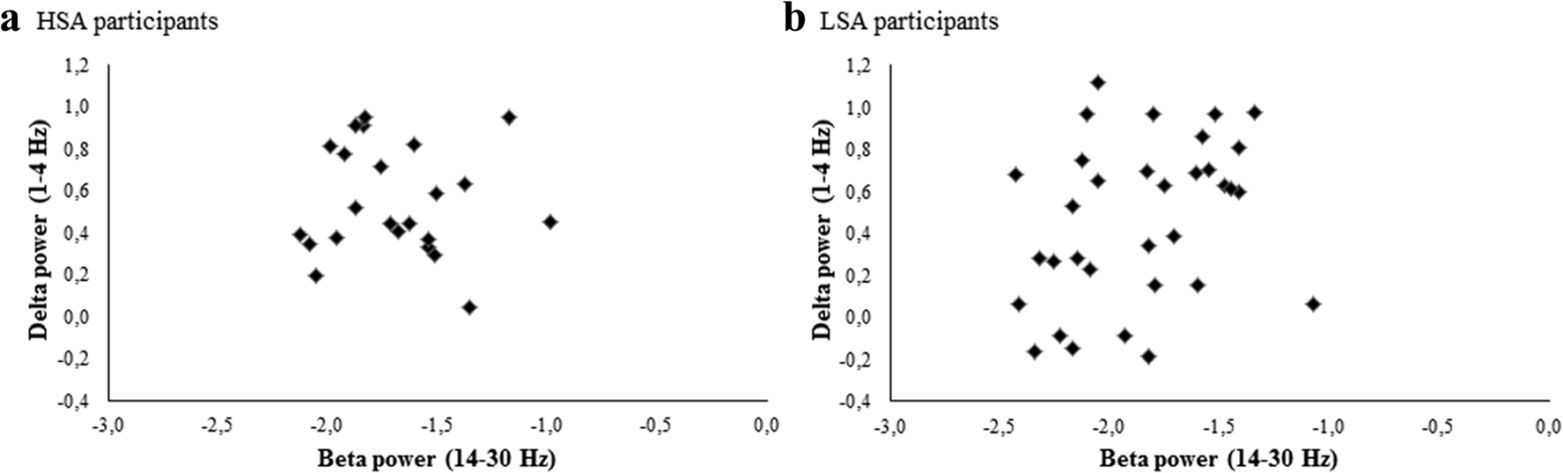
Scatterplots of relative total beta and relative delta power in HSA and LSA participants during resting state

As we hypothesized, delta–beta correlation did not differ between HSA and LSA participants during the resting state (HSA *τ* = –.01, LSA *τ* = .23; *Z* = .87, *p* = .38) (Fig. 5). Similar results were found for the within-subjects measure of delta–beta correlation (see Supplementary Data 2).

#### Social performance task

**Fig. 6:**
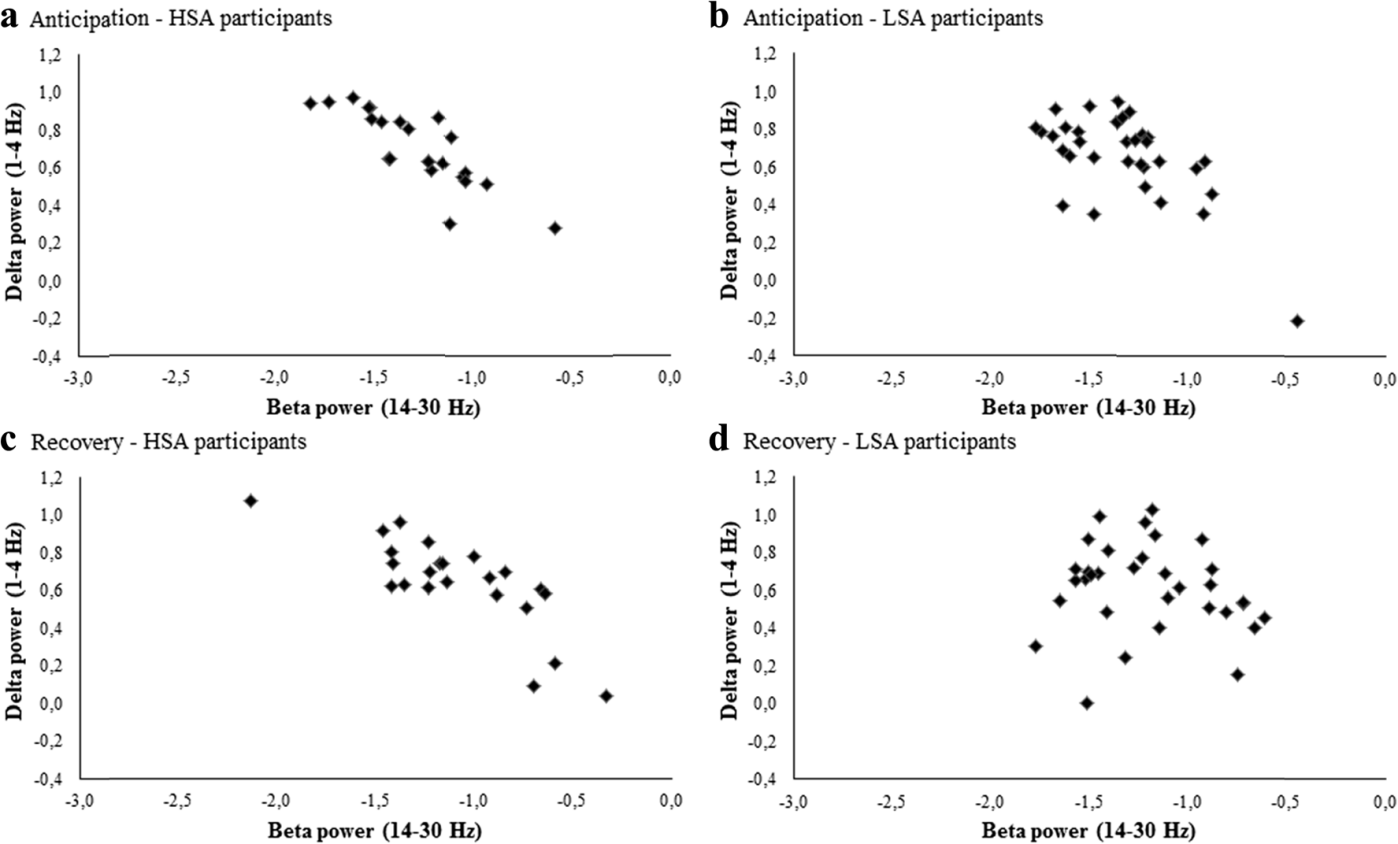
Scatterplots of relative total beta and relative delta power in HSA and LSA participants during the social performance task

First, we examined group differences in delta–beta correlation during anticipation and recovery (Table 3). During the anticipation phase, delta–beta correlation was significantly more negative in HSA (*τ* = –.76) than in LSA (*τ* = –.39) participants, *Z* = 2.07, *p* = .04. During the recovery phase, delta–beta correlation was also significantly more negative in HSA (*τ* = –.61) than in LSA (*τ* = –.13) participants, *Z* = 1.98, *p* = .05 (Fig. 6). However, when we used a Bonferroni correction, these findings were not significant. Notably, similar analyses performed on the within-subjects measure of delta–beta correlation yielded significantly more negative correlations during the anticipation phase, relative to the recovery phase, in both groups (see Supplementary Data 2).

To examine more closely whether the findings above were driven by either low or high beta frequencies, the analyses were run for low (14–20 Hz) and high (20–30 Hz) beta power separately. As is shown in Table 4, cross-frequency correlations between delta and either low or high beta did not differ between HSA and LSA participants.

**Table 4:** Correlations between delta and beta (also separately for low and high beta) per condition per group

		*τ*		*Z*	*p* Value
		HSA	LSA		
Anticipation	Total beta	–.76	–.39	2.07	.04
	Low beta	–.44	–.34	.42	.67
	High beta	–.67	–.31	1.67	.10
Recovery	Total beta	–.61	–.13	1.98	.05
	Low beta	–.33	–.15	.65	.52
	High beta	–.48	–.004	1.79	.07

## Discussion

The goal of the present study was to validate whether frontal alpha asymmetry and delta–beta correlation during resting state and a social performance task are putative electrophysiological measures of social anxiety. We used a social performance task to measure EEG activity during anticipation of and recovery from a stressful social situation. At the behavioral level, results showed that HSA participants were more nervous during the social performance task and felt less like doing the anticipation than LSA participants. For frontal alpha asymmetry, no significant differences were found between HSA and LSA participants during resting state, anticipation, and recovery. Delta–beta correlation differed between HSA and LSA participants. That is, HSA participants displayed enhanced negative delta–beta correlation as compared to LSA participants during anticipation of and recovery from a social performance task. This study is the first to directly compare frontal alpha asymmetry and delta–beta correlation, and shows that delta–beta correlation is a putative EEG measure of social anxiety.

During resting state, frontal alpha asymmetry did not differ between the HSA and LSA participants in our study, which is in line with our hypothesis and with previous results in patients with SAD versus controls (Davidson et al., 2000), HSA versus LSA participants (Beaton et al., 2008), and high versus low socially withdrawn participants (Cole et al., 2012). Several other findings contradict these results. For example, Moscovitch et al. (2011) found that patients with SAD showed relatively more left frontal cortical activity after cognitive–behavioral treatment, although they did not test whether these patients showed relatively increased right frontal cortical activity before treatment. Furthermore, shyness was related to frontal alpha asymmetry during resting state (Beaton et al., 2008; Schmidt, 1999). This relation with frontal alpha asymmetry was only found for shyness, not for social anxiety (Beaton et al., 2008). Because shyness might be part of the SAD spectrum (Stein, Ono, Tajima, & Muller, 2004), we expected to find the same results in these groups as in HSA participants. This might indicate that shyness and social anxiety are separate constructs: shyness is a more general discomfort in novel social situations (Henderson, 2010), whereas social anxiety is related to fear of negative evaluation (APA, 2013). If this is indeed the case, the effects of shyness cannot be generalized to social anxiety. On the basis of the present findings and prior inconsistencies in the frontal alpha asymmetry literature, we suggest that frontal alpha asymmetry is possibly not a stable EEG measure of social anxiety.

During the anticipation and recovery phases of the social performance task, frontal alpha asymmetry did not differ between HSA and LSA participants. With regard to the anticipation phase, our present results corroborate previous findings. That is, Beaton et al. (2008) demonstrated that frontal alpha asymmetry did not differ between HSA versus LSA participants during a speech preparation task. Furthermore, Cole et al. (2012) have shown that high socially withdrawn individuals did not differ in frontal alpha asymmetry from low socially withdrawn individuals when they watched a benign movie. However, after watching an anxious video, a significant increase in right frontal cortical activity was observed in high socially withdrawn participants. Probably, our results on anticipatory activity in the social performance task can best be compared with the benign condition in the Cole et al. study, because our confederate did not talk in an embarrassed and anxious way (see Supplementary Data 1). Our present findings are in contrast with those of Davidson et al. (2000), who showed that patients with SAD could be characterized by relatively increased right frontal cortical activity relative to controls when anticipating a public speech. These contradicting findings could possibly be related to the degree of anxiety elicited by the task. That is, our social performance task elicited a considerable level of nervousness in both HSA and LSA participants, whereas the social-performance task used in Davidson et al.’s study only elicited feelings of anxiety in patients with SAD. Thus, in contrast to the Davidson et al. study, our social performance task elicited higher levels of psychological arousal in the control sample, which may have resulted in deflated group differences at the electrophysiological level. During recovery, Davidson et al. showed no differences between patients with SAD and controls, which was confirmed by our results. In conclusion, it remains unclear whether frontal alpha asymmetry during a social performance task is related to social anxiety.

These mixed results in previous studies on frontal alpha asymmetry as a putative EEG measure of social anxiety could have been due to the alleged influence of depression on alpha asymmetry levels. Indeed, Thibodeau, Jorgensen, and Kim (2006) hypothesized that frontal alpha asymmetry in anxiety might be explained by comorbid depression, because effect sizes of frontal alpha asymmetry studies were near zero in samples that included anxious participants without comorbid depression. Unfortunately, most studies on frontal alpha asymmetry in social anxiety do not report comorbid depression. Future studies should compare HSA participants with high and low comorbid depression to disentangle the effects of social anxiety and depression on frontal alpha asymmetry.

The second putative EEG measure that we examined here was delta–beta correlation. As hypothesized, delta–beta correlation did not differ between HSA and LSA participants during the resting state. Indeed, delta–beta correlation is generally only increased in response to anxiogenic situations (Knyazev, 2011; Knyazev, Schutter, & van Honk, 2006), and not during the resting state in HSA and LSA participants (Miskovic et al., 2010), although children with a parent with SAD showed more delta–beta correlation during the resting state than did children with healthy parents (Miskovic, Campbell, et al., 2011). Furthermore, patients with SAD showed significant delta–beta correlation before cognitive–behavioral treatment (Miskovic, Moscovitch, et al., 2011). Taken together, there is mixed evidence that delta–beta correlation during resting state is an EEG measure of social anxiety.

During the anticipation phase of the social performance task, we found enhanced negative delta–beta correlation in HSA relative to LSA participants. This is in line with findings of Miskovic et al. (2010) and Miskovic, Moscovitch, et al. (2011) who also found a difference between, respectively HSA and LSA participants, and patients with SAD and LSA participants. They found increased positive delta–beta correlation that was accompanied by more nervousness, less confidence, less calmness, les preparedness, and poorer estimates of the anticipated speech performance (Miskovic et al., 2010). Interestingly, LSA participants also showed increased negative delta–beta correlation during anticipation versus resting state in our study. This could be related to increased nervousness, but this should be confirmed by future research. During recovery, we found enhanced negative delta–beta correlation in HSA relative to LSA participants. It seemed that HSA participants showed a prolonged reaction to the social performance task, whereas the reaction of LSA participants went back to baseline. However, these findings should be interpreted with caution, since our sample size was modest, the findings were not significant after Bonferroni correction, and delta–beta correlation was a between-subjects measure, so that we cannot draw any conclusions on the within-subjects level. No previous studies of social anxiety had measured delta–beta correlation during recovery. To summarize, this and previous studies have shown that delta–beta correlation during anticipation and recovery are putative EEG measures of social anxiety, but this should be confirmed by future research.

We found negative delta–beta correlations, whereas previous studies have shown positive delta–beta correlations (Miskovic et al., 2010; Miskovic, Campbell, et al., 2011; Miskovic, Moscovitch, et al., 2011). Typically, delta–beta correlation is interpreted as reflecting the crosstalk between cortical and subcortical brain regions (Schutter & Knyazev, 2012), where significant positive delta–beta correlation would indicate stronger functional coherence between cortical and subcortical regions (Putman, 2011). Nonsignificant delta–beta correlation is interpreted as the absence of functional coherence between cortical and subcortical regions (Miskovic & Schmidt, 2009; Schutter & Knyazev, 2012). Following this line of reasoning, significant negative delta–beta correlation would also indicate stronger functional coherence, only in a different direction. General models of anxiety suggest that increased activity in the amygdala and decreased activity in the prefrontal cortex bias the brain toward threat-related responses (Bishop, 2007). This inverse relationship between cortical and subcortical brain regions suggests a negative correlation between oscillations that stem from these regions, as found in our study.

The positive delta–beta correlation observed in previous studies could have been due to differences in the power measures calculated. Notably, previous studies have not specified which EEG power measure (i.e., absolute or relative) has been used. In the present study, we calculated relative EEG power because it better reflects cortical activity and is less confounded by scalp thickness and electrical resistance (Allen et al., 2004; Cook et al., 1998; Knyazev, 2007; Knyazev et al., 2004). It should be noted that when analyzing the delta–beta correlation using absolute power, we did obtain positive correlations; however, no differences between groups were found. Thus, the robustness of our present effects using relative power should be tested in future studies. Another possible reason for the unexpected negative delta–beta correlation could be that the relationship between delta–beta correlation and stress is not linear, but U-shaped. It is possible that at a certain level of stress the relation between delta and beta power changes. Our social-performance paradigm might have been more stressful, because LSA participants also showed increased nervousness during the task. As a result, our study might have passed the threshold that resulted in negative delta–beta correlation. Future research with different, increasingly stressful phases should give more insight into the relation between delta–beta correlation and stress.

Despite the strength of comparing frontal alpha asymmetry and delta–beta correlation during resting state, anticipation, and recovery in the same sample, this study has a few limitations that should be taken into account. First, we tested a modest sample that consisted only of female participants, which limits generalizing the present findings. Second, delta–beta correlation was computed as a between-subjects measure, to compare the findings with previous studies using the same measure (Miskovic et al., 2010; Miskovic, Campbell, et al., 2011; Moscovitch et al., 2011). However, this warrants caution with interpretation of delta and beta power within subjects. When we analyzed the data in a within-subjects way (see Supplementary Data 2), the data showed the same pattern, but the differences between HSA and LSA participants were not significant. Knyazev (2011) compared between-subjects and within-subjects measures of delta–beta correlation and concluded that these measures were similar. However, the between-subjects analysis revealed more significant results than the within-subjects analysis (Knyazev, 2011). This could be related to a difference in power between the two types of analysis, which could also explain the differences in the present study. Third, EEG during resting state was measured with eyes closed, whereas EEG during anticipation and recovery was measured with the eyes open. This allowed us to compare our findings with existing studies on social anxiety—which reported results on eyes-open task data. A potential drawback is that it interfered with direct comparison of the EEG resting-state data and data from the social performance task within this study. Indeed, it has been shown that EEG oscillations differ between eyes-open and eyes-closed resting-state conditions (Barry, Clarke, Johnstone, Magee, & Rushby, 2007). Future studies should measure resting state, anticipation, and recovery in the same way, to allow direct comparisons of EEG oscillatory power during these phases. Fourth, social anxiety has a high comorbidity with other anxiety disorders, depression and substance abuse disorders (Rapee & Spence, 2004). As we previously noted, comorbid depression could influence the relation between social anxiety and frontal alpha asymmetry. Besides obtaining reliable electrophysiological measures of social anxiety, future research should preferably focus on the specificity of such measures for social anxiety. Such specificity may have important consequences for characterization of biomarkers, as well as the development of treatment procedures of SAD.

In conclusion, the present study provided a detailed characterization of frontal alpha asymmetry and delta–beta correlation as putative EEG measures during resting state and a social performance task in HSA and LSA participants. Our results suggest that delta–beta correlation during anticipation of and recovery from a social performance task (i.e., giving a speech in front of a camera) is a putative electrophysiological measure of social anxiety. Moreover, by including both resting state, as well as task-related EEG data we were able to demonstrate that a certain level of stress might be needed to find EEG measures of social anxiety.

## Electronic supplementary material

Below is the link to the electronic supplementary material.

Supplementary Data 1 (DOCX 56 kb)

Supplementary Data 2 (DOCX 50 kb)

## Footnotes

1 An average reference scheme was employed (1) to allow for better comparison of the present data with results previously obtained in HSA samples (Cole et al., 2012; Davidson et al., 2000), and (2) because the average reference scheme best approximates an inactive reference, provided that a large array of electrodes is used (Allen et al., 2004). Furthermore, Allen et al. demonstrated that beta power shows a negative relation to brain activity when a linked-ears reference was used. The results of the present study remained the same when we used an average of the mastoids as a reference scheme.

2 We examined whether the delta power measure was confounded by arterial activity, by running an independent component analysis for a random selection of ten participants, and by extracting the electrocardiogram (ECG) component from the EEG data. These results showed that this ECG-corrected delta measure did not differ from the uncorrected delta power measure (for both relative and absolute power during the resting state for electrodes F3, F4, and Fz; all *p*s > .05), and thus the delta power uncorrected for ECG activity is reported here.

3 We found no correlations between the EEG measures (alpha asymmetry, delta, beta, low-beta, and high-beta power during resting state, anticipation, and recovery), task-related nervousness and avoidance, and questionnaire scores after correction for multiple comparisons (240 correlations, *α* = .0002).
