# Supplementary material for: Putative EEG measures of social anxiety: Comparing frontal alpha asymmetry and delta–beta cross-frequency correlation
Source: Cogn Affect Behav Neurosci. 2016 Aug 24;16(6):1086–98. doi: 10.3758/s13415-016-0455-y (PMC5153416; doi:10.3758/s13415-016-0455-y)
Supplement: Supplementary file 1 — (DOCX 56 kb) [file 13415_2016_455_MOESM1_ESM.docx]

**Supplementary data 1**

**Ratings of the video and expectations of own speech**

After participants viewed the video of a peer, they indicated how socially competent, attractive and nervous the person on the video was, and whether they would like to meet the person. Right before participants had to give their own speech, we asked them to indicate how they expected to be judged by another person on the same four questions (see supplementary figure 1). For social competence, all participants judged the other as more socially competent than they expect to be judged themselves, *F*(1, 54) = 62.45, *p* < .001, *partial η^2^* = .54, and HSA participants showed overall lower ratings, *F*(1, 54) = 9.04, *p* = .004, *partial η^2^* = .14. Furthermore, there was a significant interaction effect between group and ratings, *F*(1, 54) = 14.26, *p* < .001, *partial η^2^* = .21, indicating that HSA participants expected to be judged as less socially competent than LSA participants expected. For attractiveness, there was only a significant interaction effect between group and ratings, *F*(1, 54) = 7.10, *p* = .01, *partial η^2^* = .12, suggesting that HSA participants expected to be judged as less attractive than LSA participants expected. No main effects were found for attractiveness, all *p*s > .05. For nervousness, there was only a significant main effect of rating, *F*(1, 54) = 102.96, *p* <.001, *partial η^2^* = .66, indicating that all participants judged the peer as less nervous than they expected to be judged themselves. There was no effect of group and no interaction effect, all *p*s > .05. For the question ‘Would you like to meet this person’, there was only a significant interaction between group and ratings, *F*(1, 54) = 6.15, *p* = .02, *partial η^2^* = .10, showing that HSA participants would like to meet the peer more than that they expected that a peer would like to meet them, whereas LSA participants would like to meet the peer less than that they expected that a peer would like to meet them. There were no main effects, all *p*s > .05.


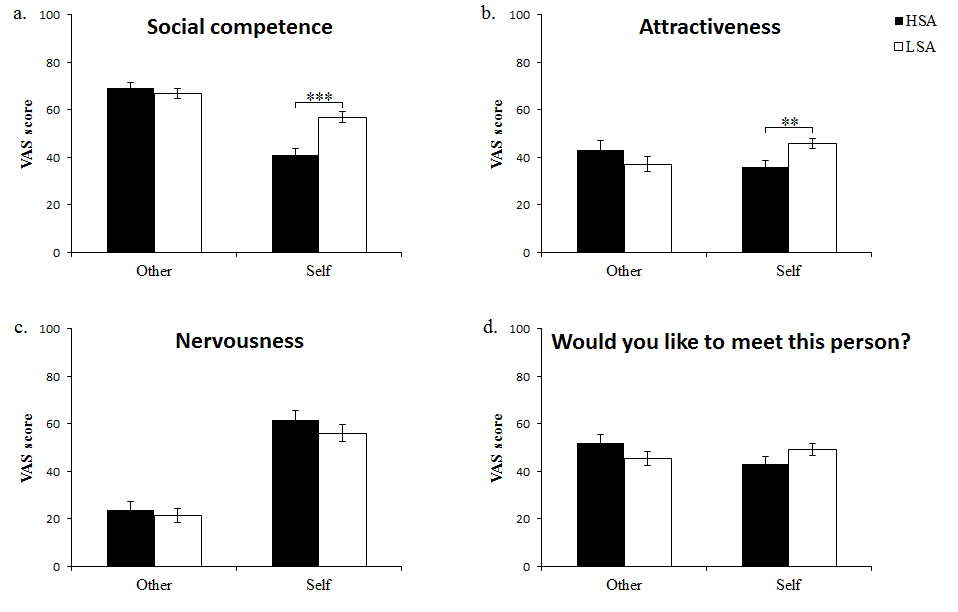


Supplementary figure 1. VAS scores of ratings of other (after viewing video) and self (before giving speech) on four questions for HSA and LSA participants (*** *p* < .001 ** *p* < .01 * *p* < .05, error bars represent standard error).
