# Supplementary material for: Putative EEG measures of social anxiety: Comparing frontal alpha asymmetry and delta–beta cross-frequency correlation
Source: Cogn Affect Behav Neurosci. 2016 Aug 24;16(6):1086–98. doi: 10.3758/s13415-016-0455-y (PMC5153416; doi:10.3758/s13415-016-0455-y)
Supplement: Supplementary file 2 — (DOCX 50 kb) [file 13415_2016_455_MOESM2_ESM.docx]

**Supplementary data 2**

**Within-subjects delta-beta correlation**

We also extracted relative delta (1-4 Hz), total beta (14-30 Hz), low beta (14-20 Hz) and high beta (20-30 Hz) power per epoch, and calculated per participant the correlation between log-transformed delta power and log transformed total, low, or high beta power.

During resting, there was no difference in within-subject delta-beta correlation between HSA and LSA participants, *F* (1, 54) = 0.28, *p* = .60, *η^2^* = .01, *F* (1, 54) = 1.23, *p* = .27, *η^2^* = .02, *F* (1, 54) = 0.07, *p* = .80, *η^2^* = .001 (respectively total, low, and high beta, see supplementary figure 2).

Supplementary figure 2. Within-subject correlation between relative delta and total beta

power for HSA and LSA participants during resting state (error bars represent standard error of the mean).

During the social performance task, there was also no difference between HSA and LSA participants, *F* (1, 54) = 1.46, *p* = 0.23, *partial η^2^* = .03, *F* (1, 54) = 2.72, *p* = .11, *partial η^2^* = .05, *F* (1, 54) = 0.81, *p* = .37, *partial η^2^* = .02 (respectively total, low, and high beta). The interaction between Time (anticipation, recovery) and Group (HSA, LSA) was not significant, *F* (1, 54) = 0.36, *p* = .55, *partial η^2^* = .01, *F* (1, 54) = 0.13, *p* = .72, *partial η^2^* = .002, *F* (1, 54) = 1.61, *p* = .21, *partial η^2^* = .03 (respectively total, low, and high beta). Within-subject delta-beta correlation was more negative during anticipation than recovery for total beta, *F* (1, 54) = 7.19, *p* = .01, *partial η^2^* = .12, but not for low and high beta, respectively *F* (1, 54) = 0.58, *p* = .45, *partial η^2^* = .01, *F* (1, 54) = 3.66, *p* = .06, *partial η^2^* = .06 (see supplementary figure 3).


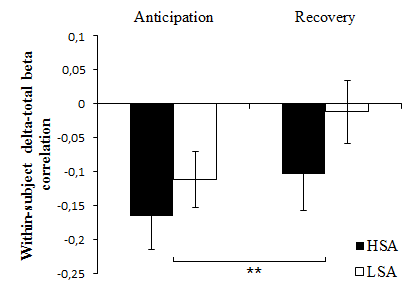


Supplementary figure 3. Within-subject correlation between relative delta and total beta power for HSA and LSA participants during the social performance task (** *p* < .01, error bars represent standard error).
